# Differential transcriptional regulation of hypoxia-inducible factor-1α by arsenite under normoxia and hypoxia: involvement of Nrf2

**DOI:** 10.1007/s00109-016-1439-7

**Published:** 2016-06-10

**Authors:** Zukaa al Taleb, Andreas Petry, Tabughang Franklin Chi, Daniela Mennerich, Agnes Görlach, Elitsa Y. Dimova, Thomas Kietzmann

**Affiliations:** 1 Faculty of Biochemistry and Molecular Medicine, Biocenter Oulu, University of Oulu, Aapistie 7, FI-90220 Oulu, Finland; 2 Experimental and Molecular Pediatric Cardiology, German Heart Center Munich, Technical University Munich, Munich, Germany; 3 DZHK (German Centre for Cardiovascular Research), partner site Munich Heart Alliance, Munich, Germany

**Keywords:** Hypoxia-inducible factor 1 (HIF-1α), Arsenite As(III), Mitogen-activated protein kinase (MAPK), Reactive oxygen species (ROS), NADPH enzyme oxidase 4 (NOX4)

## Abstract

**Abstract:**

Arsenite (As(III)) is widely distributed in nature and can be found in water, food, and air. There is significant evidence that exposure to As(III) is associated with human cancers originated from liver, lung, skin, bladder, kidney, and prostate. Hypoxia plays a role in tumor growth and aggressiveness; adaptation to it is, at least to a large extent, mediated by hypoxia-inducible factor-1α (HIF-1α). In the current study, we investigated As(III) effects on HIF-1α under normoxia and hypoxia in the hepatoma cell line HepG2. We found that As(III) increased HIF-1α protein levels under normoxia while the hypoxia-mediated induction of HIF1α was reduced. Thereby, the As(III) effects on HIF-1α were dependent on both, transcriptional regulation via the transcription factor Nrf2 mediated by NOX4, PI3K/Akt, and ERK1/2 as well as by modulation of HIF-1α protein stability. In line, the different effects of As(III) via participation of HIF-1α and Nrf2 were also seen in tube formation assays with endothelial cells where knockdown of Nrf2 and HIF-1α abolished As(III) effects. Overall, the present study shows that As(III) is a potent inducer of HIF-1α under normoxia but not under hypoxia which may explain, in part, its carcinogenic as well as anti-carcinogenic actions.

**Key message:**

- As(III) increased HIF-1α under normoxia but reduced its hypoxia-dependent induction.
- The As(III) effects on HIF-1α were dependent on ROS, NOX4, PI3K/Akt, and ERK1/2.
- The As(III) effects under normoxia involved transcriptional regulation via Nrf2.
- Knockdown of Nrf2 and HIF-1α abolished As(III) effects in tube formation assays.
- The data may partially explain As(III)’s carcinogenic and anti-carcinogenic actions.

## Introduction

Several regions in Argentina, Canada, India, Japan, China, Taiwan, Mexico, Chile, and the USA are characterized by the presence of inorganic arsenic as natural contaminant in drinking water. This implies that more than 100 million persons are chronically exposed to inorganic arsenic at levels higher than the maximum contaminant level of 0.13 μmol/l set by the WHO [1].

It is long known that chronic arsenic inhalation or ingestion is toxic and causes a number of health problems. Although the clinical manifestations of arsenic toxicity vary widely between persons and populations, chronic exposure to arsenic is, if not causing death, associated with a greater risk of ischemic heart disease [2], neurologic consequences [3] in adults and children [4] as well as with the appearance of cancer [5] in the lung, skin, kidney, bladder, and liver [6].

Solid tumors are characterized by the occurrence of areas with limited oxygen supply and it was shown that cells present in the hypoxic areas contain high levels of the hypoxia-inducible factor-1 alpha protein (HIF-1α) (for review, see [7]). The HIF-1α protein forms, together with its heterodimeric partner HIF-1β or ARNT, the proper transcription factor hypoxia-inducible factor-1 (HIF-1) which has been found to be critical for the regulation of more than 100 genes in response to hypoxia. A prerequisite for the formation of the HIF-1 heterodimer is the hypoxia-dependent stabilization of the HIF-1α subunit and the recruitment of coactivators. This is primarily achieved at the post-translational level by hydroxylation events occurring at the N-terminal (N-TAD) and C-terminal (C-TAD) transactivation domain of HIF-1α [7–9]. Further, phosphorylation events [10–12] and mechanisms leading to enhanced HIF-1α gene transcription [13] also contribute to an increase in HIF-1α protein levels. In addition to hypoxia, HIF-1α is also responsive to a variety of non-hypoxic stimuli among them transition metals such as CoCl_2, _chromium, or arsenite (for review, see [14, 15]).

Although arsenite seems to exert some of its effects through direct interaction of the trivalent arsenite anion (AsO_3_^3–^) with the thiol groups of vicinal cysteines in target proteins [16, 17], the underlying mechanisms by which arsenic contributes to HIF-1α activation and carcinogenesis are rather controversial [18–20] and thus remain largely unknown. In particular, the effects of arsenite on the regulation of HIF-1α under tumor-promoting conditions like hypoxia have never been explored. In order to gain a better understanding about the mechanisms and actions involved when cells are exposed to arsenite, we focused in the current study on the impact of arsenite on HIF-1α regulation under normoxia and hypoxia.

We found that arsenite induced the levels of HIF-1α under normoxia via a transcriptional mechanism involving NADPH oxidase-generated reactive oxygen species (ROS), the PI3K/Akt and ERK1/2 pathway, and the redox-dependent transcription factor Nrf2. Moreover, arsenite was able to induce DNA synthesis and angiogenesis in a Nrf2- and HIF-1α-dependent manner. By contrast, arsenite reduced the hypoxia-dependent upregulation of HIF-1α, though increasing its stability. Together, the current findings indicate that arsenite may act as a powerful modulator of the HIF system with different effects under normoxia and hypoxia which may explain, in part, its ambivalent role in cancer and its potential therapeutic use.

## Materials and methods

All biochemicals and enzymes were of analytical grade and purchased from commercial suppliers. Sodium arsenite (As(III)), actinomycin D (ActD), and cycloheximide (CHX) were purchased from Sigma. The specific inhibitors for the mitogen-activated protein kinase (MEK) U0126 and PD98059, the phosphatidylinositol 3-kinase (PI3K) inhibitor LY294002, Jun N-terminal kinase (JNK) inhibitor SP600125, and the p38 protein kinase inhibitor SB202190 were purchased from Enzo, Roche Diagnostics GmbH (Mannheim).

### Cell culture and cell treatment

Human hepatoma HepG2 cells were cultured in a normoxic atmosphere at 16 % O_2_, 79 % N_2_, and 5 % CO_2_ (by volume) in Earle’s minimal essential medium supplemented with 10 % fetal calf serum (FCS) for 24 h. At 24 h, the medium was changed and the culture was continued under normoxic conditions at 16 % O_2_ or hypoxic conditions at 5 % O_2_ (with 90 % N_2_ and 5 % CO_2_ [by volume]).

Human microvascular endothelial cells (HMEC-1) were cultured under the same atmospheres in MCDB131 medium (Gibco) supplemented with 10 % FCS. Nrf2 knockdown mouse embryonic fibroblasts were cultured in Dulbecco’s modified Eagle’s medium (DMEM) with 10 % fetal calf serum and were a kind gift from S. Immenschuh.

For treatment of cells with As(III), cells were cultured in 60 mm diameter dishes and grown overnight. Thereafter, As(III) was added to the media, and cells were further incubated for the time periods indicated. None of the used As(III) concentrations exerted unspecific cell damage or toxicity as assessed by LDH leakage (data not shown). Pre-treatment of cells with kinase inhibitors, ActD, and CHX was performed for 30 min, respectively, prior to the addition of As(III).

### Plasmid constructs

The reporter constructs for pGL3-EPO-HRE [21], p(ARE)-p44-Luc [22], as well as siSTRIKE ™ U6 shRNA Nrf2, siSTRIKE ™ U6 shRNA HIF-1α plasmids, siSTRIKE ™ U6 shNOX4 were described previously [23, 24].

### RNA preparation and Northern blot analysis

The isolation of total RNA and Northern blot analysis were performed as described previously [21]. Digoxigenin-labeled antisense RNAs hybridization probes were generated by in vitro transcription from pBS-Actin by using T7 polymerase or from pCRII-HIF-1α and pBS-HO-1 by using T7 polymerase. Blots were quantified with a video densitometer (Biotech Fischer, Reiskirchen, Germany).

### Western blot analysis

Western blot analysis was carried out as described previously [21]. In brief, 100 μg of protein from medium or total lysates from HepG2 cells was subjected to Western blotting with monoclonal antibodies against PAI-1 (1:100; American Diagnostics, Pfungstadt, Germany), HIF-1α (1:1000; BD Biosciences, Heidelberg, Germany), phospho p42/p44 MAPK (1:1000), phospho p38 MAPK (1:1000), phospho Akt-T308 (1:1000) and phospho c-Jun-Ser63-Ser73 (1:1000) (Cell Signaling) or rabbit polyclonal antibodies against HO-1 (1:1000; Cell Signaling, Biomol, Hamburg), Golgi membrane (58-kD cis-Golgi protein formiminotransferase cyclodeaminase) (1:10,000; Biosciences, Goettingen, Germany), or α-tubulin (1:10,000; Sigma). The enhanced chemiluminescence (ECL) system was used for detection (Amersham Biosciences).

### Cell transfection and luciferase assay

About 4 × 10^5^ HepG2 cells per 60 mm diameter dish were transfected as described previously [11, 22]. In brief, cells were transfected with 2 μg pGL3-EPO-HRE or p(ARE)-p44-Luc, together with 0.25 μg of a *Renilla* luciferase expression construct (pRLSV40) (Promega, Heidelberg, Germany) to control transfection efficiency. The detection of luciferase activity was performed with a luciferase assay kit (Berthold, Pforzheim, Germany).

For knockdown experiments, cells were transfected with 10 μg of the respective psiSTRIKE shRNA plasmids overnight. The next day, the medium was changed and cells were treated as described above.

### Protein half-life

Protein half-life studies were performed in HepG2 cells treated with 50 μM As(III) under normoxia and hypoxia for 4 h. After 4 h, cycloheximide (100 μg/ml) was added to the medium for different times and cells were further cultured under normoxia and hypoxia. After harvesting the cells, endogenous HIF-1α protein levels were measured by immunoblot analysis.

### Measurement of ROS

Measuring ROS was performed by using the non-fluorescent dye dichlorodihydrofluorescein diacetate (H2DCFDA). Fluorescence measurements were performed in six-well plates (10,000 cells/well). The cells were treated with 50 μM As(III) and after 4 h the cells were washed with 1× PBS and 10 μM H2DCFDA was added for 20 min at 37 °C in dark. DCF fluorescence in each well was measured with a Fluoroscan fluorescence plate reader at 37 °C at an excitation wavelength of 488 nm and an emission wavelength of 538 nm. In addition, ROS measurements were performed by electron paramagnetic resonance (EPR) using the spin probe 1-hydroxymethoxycarbonyl-2,2,5,5-tetramethylpyrrolidine hydrochloride (CMH, Noxygen, Elzach, Germany). Briefly, cells were treated with As(III) in the presence or absence of hypoxia (5 % O2) for 4 h. Cells were washed once in 1× PBS, harvested in Krebs-Hepes buffer and supplemented with 5 µM diethyldithiocarbamate, 25 µM desferrioxamine, and 100 µM CMH under normoxic or hypoxic conditions. Cell suspensions were placed in air-tight glass capillaries and spectra were recorded in an EPR spectrometer with temperature-controlled resonator (e-scan, Noxygen). EPR settings for CMH spin label were as follows: center field 3455 G, sweep width 10 G, frequency 9.7690 GHz, microwave power 23.89 mW, and modulation amplitude 2.93 G. Spectra were recorded over 10 min.

### BrdU cell proliferation and colony formation assay

HepG2 cells transfected with plasmids expressing control scrambled shRNA or shRNA against HIF-1α or Nrf2 were cultured with or without As(III) in 96-well plates for 4 h. The bromodesoxyuridine (BrdU) assay (Calbiochem) was performed according to the manufacturer’s instructions and the absorbance was measured at dual wavelengths of 450 and 540 nm.

Colony formation was assessed in a Hep3B monolayer assay by seeding cells at different densities (500–3000 cells/well in steps of 500) in six-well plates and growing them for 4 days. Afterwards, cells were fixed and stained with crystal violet counted and quantified.

### In vitro matrigel angiogenesis assay

HMEC-1 cells were transfected with expression vectors for shHIF-1α, shNrf2, or with the respective control vectors. Cells were seeded at a density of 50,000 cells/well on a 96-well plate mounted with growth factor reduced Matrigel (BD Biosciences). The cells were allowed to settle for 2 h in normoxia and were then cultured where indicated in the presence of As(III). After incubation, the cells were stained with the fluorescent dye Calcein AM (Mobitec, Germany). The formation of capillary-like structures was assessed by light microscopy and quantified using Image J software (Wright Cell Imaging Facility, Toronto, Canada).

### Statistical analysis

Densitometry data were plotted as fold induction of relative density units, with the zero value absorbance in each figure set arbitrarily to 1 or 100 %. Statistical comparisons of absorbance differences were performed by the Mann-Whitney test (Statview 4.5, Abacus Concepts, Berkeley, CA), and *p* values *p* ≤ 0.05 were considered significant. Values presented are means ± SEM. Results were compared by ANOVA for repeated Luc measurements followed by the Newman-Keuls test. A probability level *p* ≤ 0.05 was accepted as significant.

## Results

### Arsenite induces HIF-1α protein levels under normoxia but reduces the effect of hypoxia

**Fig. 1:**
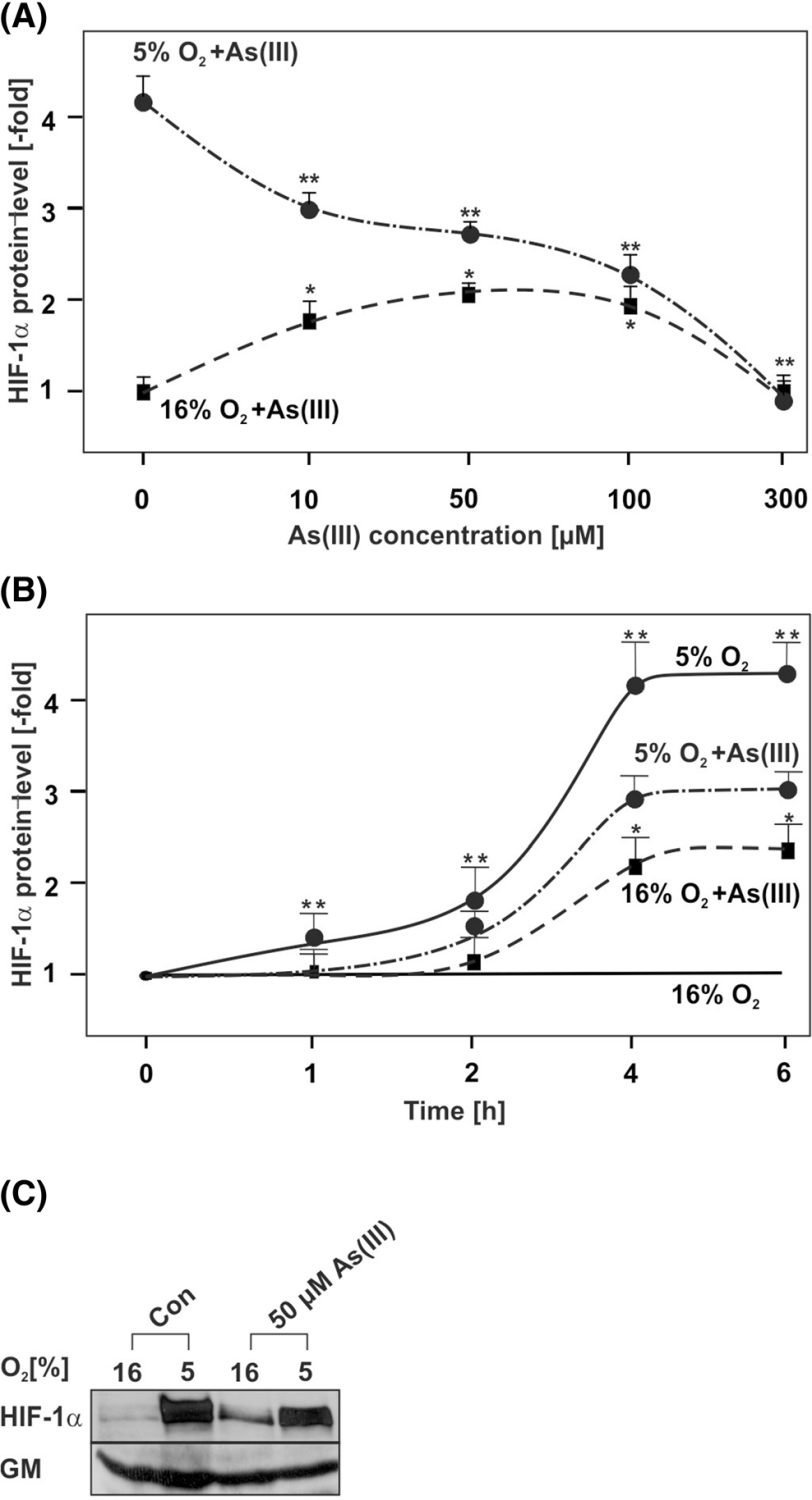
Concentration- and time-dependent modulation of HIF-1α protein levels by As(III) under normoxia and hypoxia. Cells were cultured under normoxia (16 % O_2_) for 24 h. After 24 h, the medium was changed and cells were further cultured under normoxia and hypoxia (5 % O_2_) and treated either with: **a** different concentrations of As(III) for 4 h; **b** 50 μM As(III) for the indicated time points. The HIF-1α levels in the absence of As(III) or in controls at 0 h were set to 1. Values represent mean ± SEM of at least three independent experiments. *Single asterisk*, significant difference control under normoxia vs. As(III); *double asterisks*, significant difference control under hypoxia vs. As(III). **c** Representative Western blot with 50 μM As(III) at 4 h. One hundred micrograms of total protein was subjected to Western blot analysis with an antibody against HIF-1α or Golgi membrane (*GM*). Autoradiographic signals were visualized by chemiluminescence and quantified by video densitometry

To examine the effect of As(III) on HIF-1α protein levels in HepG2 cells, different concentrations of As(III) were first applied to the cells for 4 h under normoxia and hypoxia. The western blot analyses revealed that treatment of cells with As(III) at concentrations of 10, 50, and 100 μM induced HIF-1α under normoxia (Fig. 1a, c); a higher concentration of 300 μM As(III) did not exert an effect on HIF-1α under normoxia (Fig. 1a, c). Interestingly, the inducing effect of As(III) on HIF-1α was lost when the As(III) treatment was performed under hypoxia. While hypoxia alone exerted the strongest induction on HIF-1α (about 4-fold), co-treatment with 10, 50, 100, and also 300 μM As(III) reduced the hypoxia-dependent induction of HIF-1α by about 20, 30, 50, and 100 %, respectively (Fig. 1a, c).

In addition, we determined a time course of the HIF-1α response towards As(III) under normoxia and hypoxia. The western blot analyses showed that As(III) slightly induced HIF-1α protein levels already after 2 h under normoxia. The induction increased and became maximal after 4 h; thereafter HIF-1α levels did not increase further. In addition, the results show that the hypoxia-dependent HIF-1α protein induction starts after 2 h; treatment with As(III) for 2 h and longer inhibited the hypoxia-dependent induction of HIF-1α (Fig. 1b, c). Together, these data indicate that treatment of cells with As(III) induced HIF-1α protein levels under normoxia but reduced the hypoxia-dependent HIF-1α induction.

### Arsenite regulates HIF-1α at the transcriptional level

**Fig 2:**
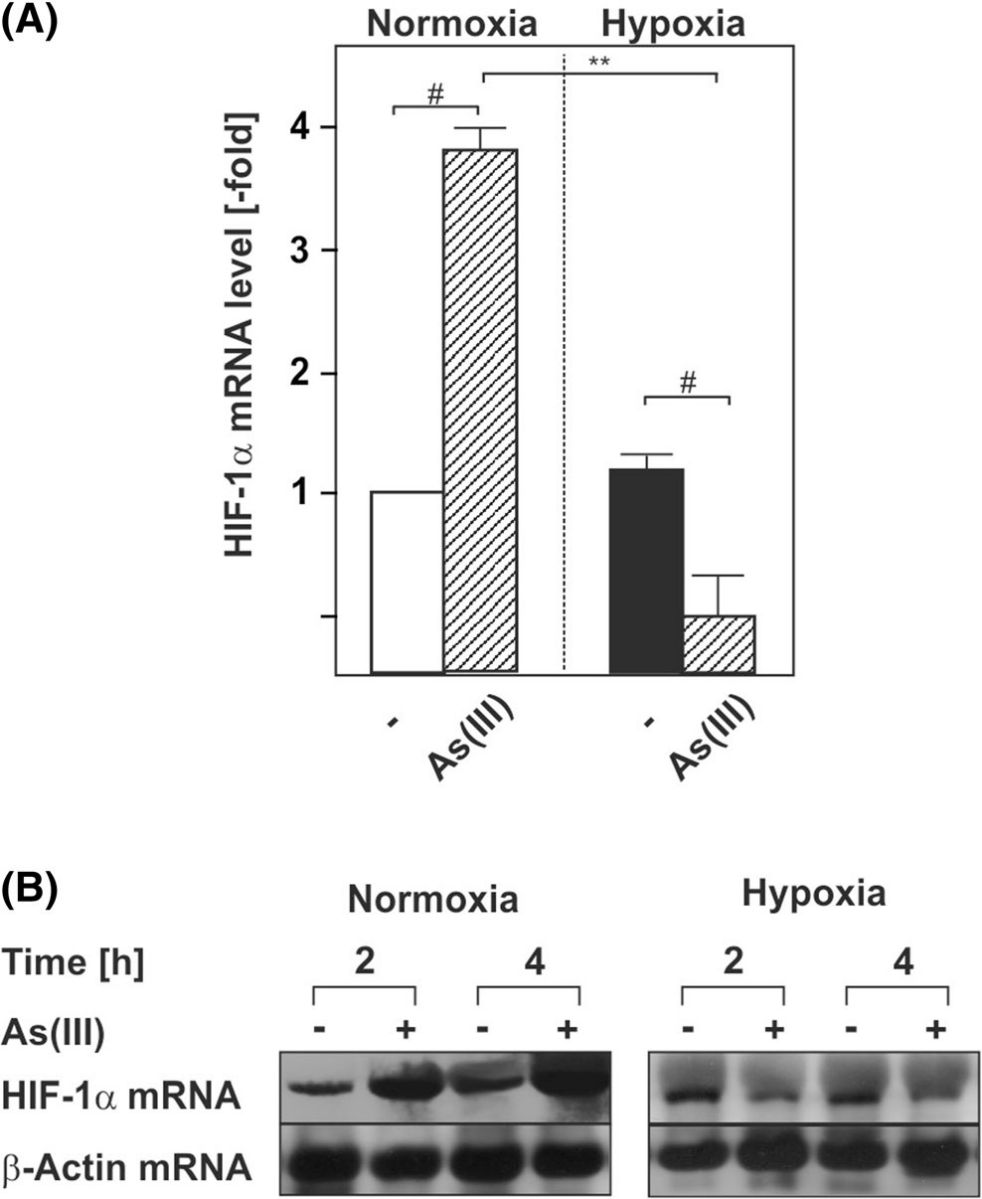
As(III) induces HIF-1α at the transcriptional level. Cells were cultured under normoxia for 24 h. After 24 h, the medium was changed and cells were further cultured under normoxia and hypoxia in the absence or presence of 50 μM As(III). **a**, **b** The HIF-1α mRNA expression levels under normoxia (16 % O_2_) were set to 1. Values represent means ± SEM of three independent experiments. *Single number sign*, significant difference As(III) vs. respective control; *double asterisks*, significant difference As(III) at 16 % O_2_ vs. As(III) at 5 % O_2_; *p* ≤ 0.05. **b** Representative Northern blot. Twenty micrograms of total RNA from cultured HepG2 cells were subjected to Northern blot analysis and hybridized with DIG-labeled HIF-1α and β-actin probes. Autoradiographic signals were visualized by chemiluminescence and quantified by video densitometry

To gain insight into the mechanisms involved, we asked whether As(III) regulates HIF-1α via transcriptional mechanisms. Initial experiments with the transcription inhibitor actinomycin D supported the notion that As(III) affects HIF-1α transcription under normoxia and hypoxia (data not shown). To further specify whether or not As(III) is able to regulate HIF-1α expression at the mRNA level, Northern blot analyses were performed. Time course experiments indicated that As(III) induced HIF-1α mRNA levels by about 4-fold within 4 h of treatment under normoxia. However, As(III) reduced HIF-1α mRNA levels by about 50 % under hypoxia (Fig. 2a, b). Together, these data provide the first evidence that As(III) is able to regulate HIF-1α at the transcriptional level.

### Arsenite induces HIF-1α synthesis under normoxia and prolongs its half-life under hypoxia

**Fig. 3:**
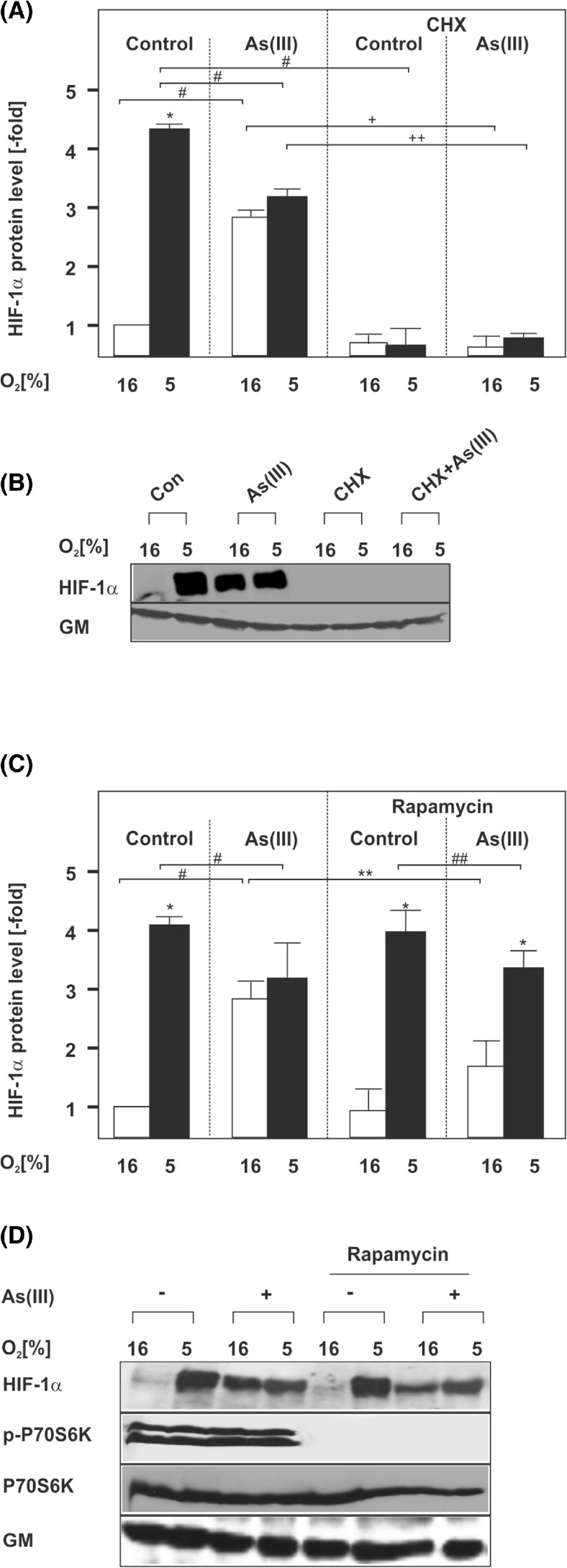
Induction of HIF-1α by As(III) is dependent on mTORC1-driven protein synthesis. Cells were cultured under normoxia for 24 h. At 24 h, the medium was changed and the cells were pre-treated for 30 min either with cycloheximide (*CHX*, 10 μg/ml) (**a**, **b**) or rapamycin (10 μM) (**c**, **d**) and then treated with 50 μM As(III) under normoxia (16 % O_2_) or hypoxia (5 % O_2_) for 4 h. **a**, **c** Statistical analyses of HIF-1α levels. The HIF-1α levels in the controls were set to 1. Values represent mean ± SEM of three independent experiments. *Single asterisk*, significant difference 16 % O_2_ vs. 5 % O_2_; *single number sign*, significant difference As(III) vs. control; *double asterisks*, significant difference 16 % O_2_ + As(III) vs. rapamycin at 16 % O_2_ + As(III); *double number sign*, significant difference rapamycin at 5 % O_2_ vs. rapamycin at 5 % O_2_ + As(III); *single plus sign*, significant difference As(III) 16 % O_2_ vs. As(III)/CHX 16 % O_2_; *double plus sign*, significant difference As(III) 5 % O_2_ vs. As(III)/CHX 5 % O_2_; *p* ≤ 0.05. **b**, **d** Representative Western blot. One hundred micrograms of isolated total protein was subjected to Western blot analysis with an antibody against HIF-1α, Golgi membrane (*GM*), phospho-p70S6K1, or total p70S6K1. Signals were visualized by chemiluminescence and quantified by video densitometry

To further analyze the mechanisms by which As(III) regulates HIF-1α levels, we next examined whether ongoing protein synthesis or post-translational effects are involved. To do this, we first used cycloheximide (CHX) as translation inhibitor. Like in the previous experiments, As(III) induced HIF-1α strongly under normoxia but reduced the hypoxia-dependent upregulation. When CHX was used, it completely abolished the induction of HIF-1α by As(III) under normoxia but also the response towards hypoxia or combined treatment (Fig. 3a, b).

Since the above data indicated the involvement of protein synthesis in the regulation of HIF-1α by As(III), we next asked whether this involves the rapamycin-sensitive mTORC1 complex. The mTORC1 activates translation via phosphorylation of p70-S6 Kinase-1 and the eukaryotic initiation factor 4E binding protein 1 (4E-BP1) which converge at the translation initiation complex at the 5′ end of the respective mRNA.

To test the participation of mTORC1 in the As(III)-mediated effects on HIF-1α, we used rapamycin as mTORC1 inhibitor. As before, treatment with As(III) under normoxia resulted in a strong upregulation of HIF-1α. Rapamycin reduced the As(III)-dependent HIF-1α induction by about 50 % under normoxia but not under hypoxia (Fig. 3c, d).

**Fig. 4:**
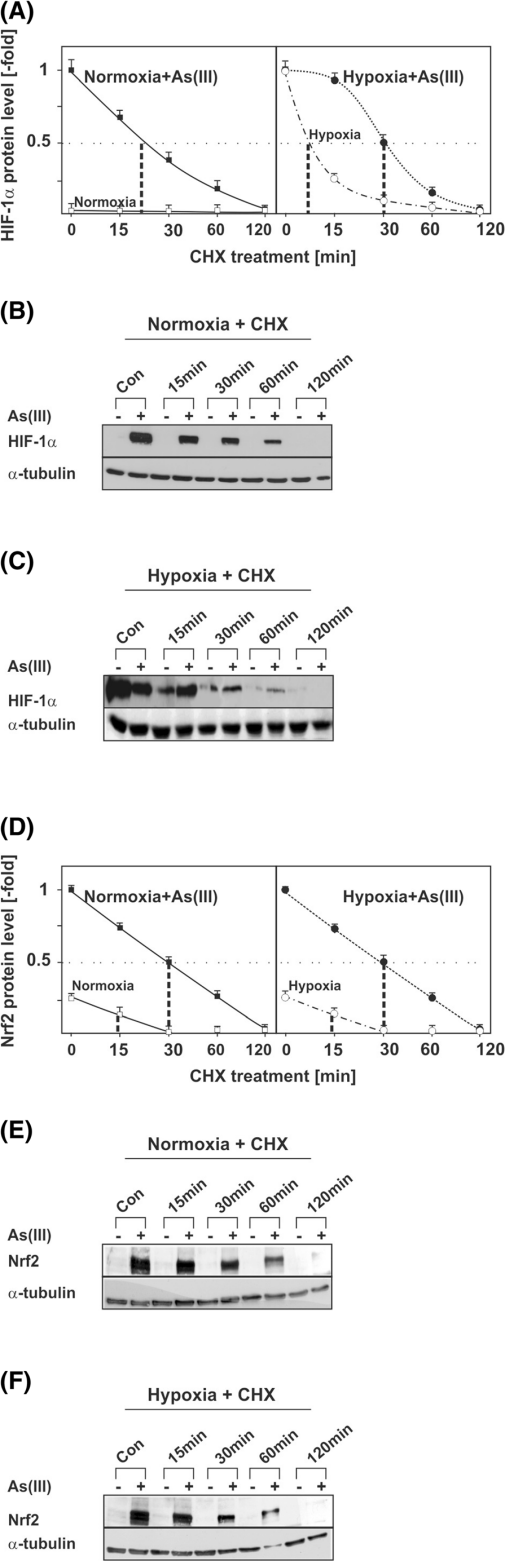
Modulation of HIF-1α and Nrf2 half-life by As(III). HepG2 cells were cultured under normoxia for 24 h. At 24 h, the medium was changed and the cells were treated with 50 μM As(III) under normoxia (16 % O_2_) or hypoxia (5 % O_2_) for 4 h. After 4 h, cells were treated with cycloheximide (*CHX*, 10 μg/ml) for the indicated times. **a** The HIF-1α levels in the As(III)-treated controls were set to 1. Values represent mean ± SEM of three independent experiments. **b**, **c** Representative Western Blots. One hundred micrograms of isolated total protein was subjected to Western blot analysis with an antibody against HIF-1α or α-tubulin. **d** The Nrf2 levels in the As(III)-treated controls were set to 1. Values represent mean ± SEM of three independent experiments. **e**, **f** Representative Western Blots. One hundred micrograms of isolated total protein was subjected to Western blot analysis with an antibody against Nrf2 or α-tubulin

These data support the notion that HIF-1α induction by As(III) under normoxia involves mTORC1-regulated protein synthesis; however, they do not exclude that As(III) may affect HIF-1α protein stability under hypoxia. Therefore, we also investigated whether As(III) has an effect on HIF-1α protein stability. The half-life measurements indicated that the HIF-1α half-life was about 20 min in As(III)-treated normoxic cells (Fig. 4a). As mentioned above, As(III) was found to reduce HIF-1α protein induction under hypoxia; however, the half-life measurements showed that the HIF-1α half-life after treatment with As(III) is prolonged in comparison to cells incubated only under hypoxia (Fig. 4c). Together, these data indicate that under normoxic conditions As(III) contributes to HIF-1α regulation primarily via induction of transcription and translation whereas under hypoxia As(III) is able to stabilize HIF-1α.

### As(III) induces HIF-1α and the HIF-target genes PAI-1 and HO-1 via Nrf2

**Fig. 5:**
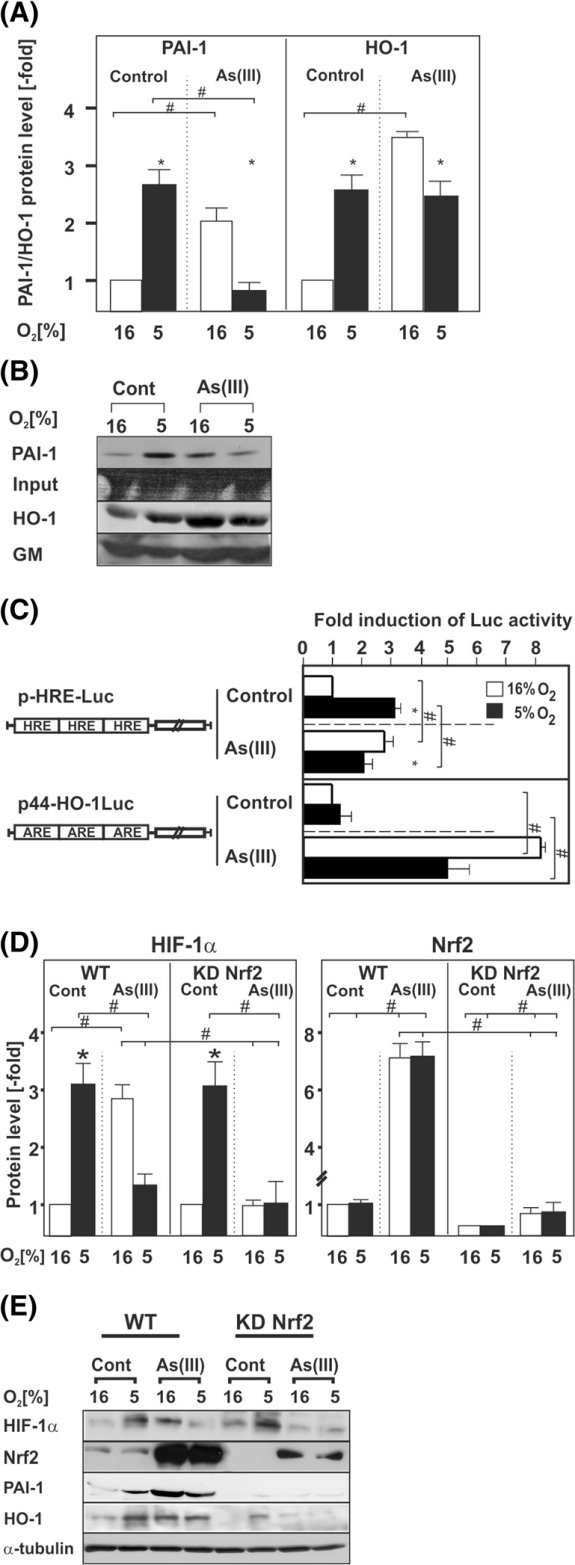
As(III) affects HIF-target gene expression and knockdown of Nrf2 abolishes As(III)-dependent HIF-1α induction. HepG2 cells were cultured under normoxia for 24 h. At 24 h, the cells were treated with 50 μM As(III) and further cultured for the next 12 h under normoxia (16 % O_2_) or hypoxia (5 % O_2_). **a** Statistical analyses of the PAI-1 and HO-1 protein levels. The PAI-1 and HO-1 levels under normoxia were set to 1. Values are means ± SEM of at least four independent experiments. *Single asterisk*, significant difference 16 % O_2_ vs. 5 % O_2_; *single number sign*, significant difference As(III) vs. control; *p* ≤ 0.05. **b** Representative Western Blot. One hundred micrograms of protein from the cell culture medium or total protein lysates was analyzed by Western blot analysis with an antibody against PAI-1, HO-1, and Golgi membrane (*GM*). **c** Cells were transfected with Luc gene constructs containing either three copies of the hypoxia-inducible factor (HIF) binding HRE element (pHRE-Luc) or the Nrf2 binding antioxidant response element (ARE) from the HO-1 gene in front of a minimal promoter (p44-HO-1Luc). The transfected cells were treated with 50 μM As(III) and further cultured for 24 h under normoxia or hypoxia. In each experiment, the LUC activity of the respective control at 16 % O_2_ was set to 1. Values are means ± SEM of three independent culture experiments; *single asterisk*, significant difference 16 % O_2_ vs. 5 % O_2_; *single number sign*, significant difference As(III) vs. control. **d** Statistical analyses of the HIF-1α and Nrf2 protein levels. Cells were cultured under normoxia for 24 h. At 24 h, the cells were treated with 50 μM As(III) and further cultured for the next 8 h under normoxia (16 % O_2_) or hypoxia (5 % O_2_). The expression of HIF-1α under normoxia was set to 1. Values are means ± SEM of at least four independent experiments. *Single asterisk*, significant difference control 16 % O_2_ vs. 5 % O_2_; *single number sign*, significant difference As(III) vs. control. **e** Representative Western Blots. One hundred micrograms of total protein was analyzed by Western blot with an antibody against HIF-1α, Nrf2, PAI-1, HO-1, and α-tubulin

In a next step, we investigated the effect of As(III) on HIF transcriptional activity and the HIF-1α target genes plasminogen activator inhibitor-1 (PAI-1) and heme oxygenase (HO-1). The results demonstrate that hypoxia, as shown earlier [25], induced PAI-1 expression by about 2.5-fold. As(III) treatment induced PAI-1 protein expression under normoxia by about 2-fold while it completely abrogated PAI-1 induction under hypoxia resembling the effects observed for HIF-1α under these conditions (Fig. 5a, b). Similar to PAI-1 and HIF-1α protein levels, we found that HIF activity was upregulated by about 3-fold under hypoxia while As(III) increased HIF activity by about 2.5-fold under normoxia and only by about 2-fold under hypoxia (Fig. 5c).

Further, HO-1 levels increased by about 2.5-fold in response to hypoxia, and by about 3.5 fold in response to As(III) under normoxia. Interestingly, the As(III) treatment under hypoxia increased HO-1 levels by 2.5-fold, similar to the situation with hypoxia alone (Fig. 5a, b). Thus, PAI-1 regulation followed the HIF-1α regulation in response to As(III) under normoxia and hypoxia, whereas the response of HO-1 towards As(III) in particular under hypoxia did not entirely parallel the response to HIF-1α suggesting an additional regulatory component.

Since HO-1 is also a bona fide target gene of the transcription factor Nrf2 (nuclear factor (erythroid-derived 2)-like 2) and well known to be responsive to As(III) [26], we also examined the response of Nrf2 towards hypoxia and As(III). Similar to HIF-1α, Nrf2 is regulated at the level of protein stability. In order to assess whether hypoxia and As(III) affect Nrf2 protein stability, we measured its half-life. In line with previous reports, we found that cellular Nrf2 is maintained at very low levels and that its half-life was about 15 min under both normoxia and hypoxia. Treatment of cells with As(III) extended the half-life of Nrf2 up to 30 min under normoxia and hypoxia (Fig. 4d–f).

Next we investigated whether hypoxia and As(III) influence Nrf2 activity. To do this, cells were transfected with a luciferase (Luc) reporter gene driven by Nrf2 binding antioxidant response elements (ARE) from the HO-1 gene in front of a minimal promoter (p44-HO-1Luc). In line with the protein half-life measurements, we found that hypoxia alone did not significantly affect Nrf2 activity. However, As(III) induced Nrf2 activity by about 8-fold under normoxia but only to about 5-fold under hypoxia (Fig. 5c). Together, these data suggest that Nrf2 could be involved in the As(III)-dependent regulation of HO-1 and likely HIF-1α.

We next asked whether a cross-talk between HIF-1α and Nrf2 exists. To test the possibility that Nrf2 could regulate HIF-1α expression, we used cells in which Nrf2 was depleted due to shRNA-mediated knockdown (Nrf2KD cells) and treated them with As(III) under normoxia and hypoxia. Similar to Nrf2 transcriptional activity, hypoxia had no effect on Nrf2 levels but As(III) strongly induced Nrf2 abundance under normoxia and hypoxia while these effects were greatly diminished in Nrf2KD cells (Fig. 5d, e).

Hypoxia and treatment with As(III) under normoxia induced HIF-1α by about 3-fold in the scrambled shRNA expressing control cells (Fig. 5d, e) as expected. Similarly, hypoxia induced HIF-1α levels also in Nrf2-depleted cells. However, As(III) was not able to induce HIF-1α, neither under normoxia nor under hypoxia (Fig. 5d, e). Concomitantly, PAI-1 and HO-1 expression showed the same pattern (Fig. 5e). Together, these data show that Nrf2 contributes to the As(III)-dependent regulation of HIF-1α levels.

### Role of ROS in As(III)-induced HIF-1α levels

**Fig. 6:**
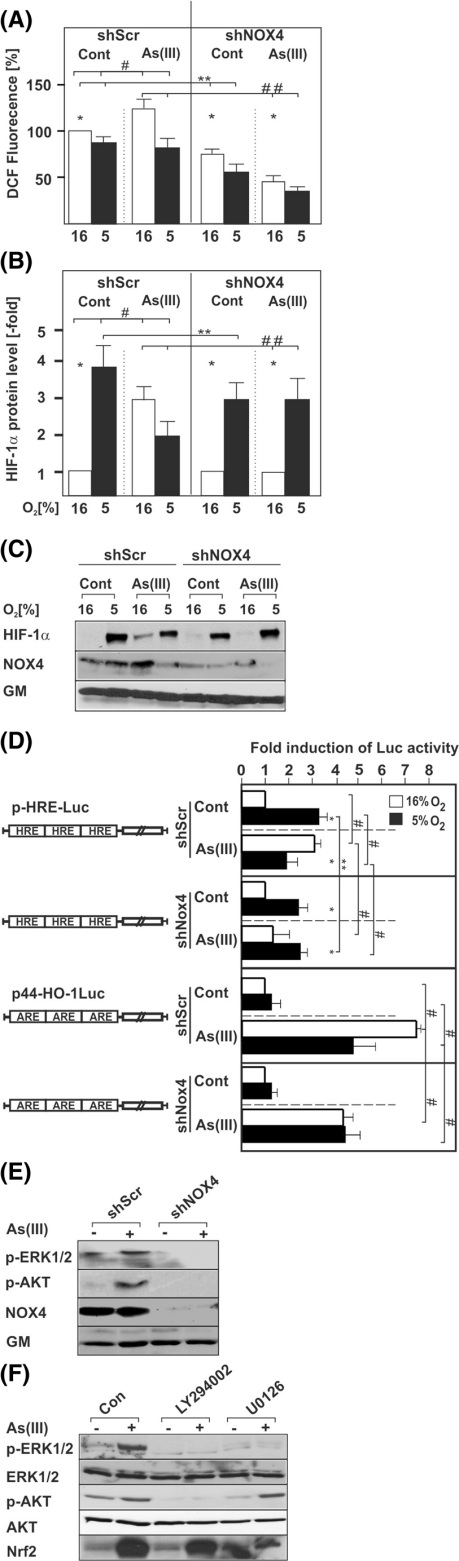
Involvement of ROS, NOX4, PI3K, and ERK1/2 in the response to As(III). Cells transfected with scrambled shRNA (shScr) or shRNAs against NOX4 (shNOX4) were cultured under normoxia for 24 h. At 24 h, the medium was changed and the cells were treated with 50 μM As(III) for 15 min under normoxia (16 % O_2_) or hypoxia (5 % O_2_). ROS formation was measured with the DCFH assay. **a** Statistical analyses of ROS formation. Values represent mean ± SEM of at least three independent experiments. *Single asterisk*, significant difference shScr vs. shNOX4; *double asterisks*, significant difference shScr vs. shScr + As(III) or shScr + As(III) vs. shNOX4 + As(III). **b** Cells transfected as above were treated with 50 μM As(III) and further cultured for 4 h under normoxia (16 % O_2_) or hypoxia (5 % O_2_). The HIF-1α protein levels in the controls under normoxia were set to 1. Values represent mean ± SEM of at least three independent experiments. *Single asterisk*, significant difference 16 % O_2_ vs. 5 % O_2_; *double asterisks*, shScr 5 % O_2_ vs. shNOX4 5 % O_2_; *double number sign*, shScr + As(III) vs. shNOX4 + As(III). **c** Representative Western blots. One hundred micrograms of total protein was subjected to Western blot analysis with an antibody against HIF-1α, NOX4, or Golgi membrane (*GM*). **d** Cells were transfected with scrambled shRNA (*shScr*) or shRNAs against NOX4 (*shNOX4*) and Luc gene constructs containing either three copies of the hypoxia-inducible factor (*HIF*) binding HRE element (*pHRE-Luc*) or the Nrf2 binding antioxidant response element (*ARE*) from the HO-1 gene in front of a minimal promoter (*p44-HO-1Luc*). The transfected cells were treated with 50 μM As(III) and further cultured for 24 h under normoxia or hypoxia. In each experiment, the LUC activity of the respective control at 16 % O_2_ was set to 1. Values are means ± SEM of three independent culture experiments; *single asterisk*, significant difference 16 % O_2_ vs. 5 % O_2_; *double asterisks*, shScr 5 % O_2_ vs. shNOX4 5 % O_2_; *single number sign*, shScr vs. shScr + As(III) or shNOX4 + As(III). **e** Cells transfected with scrambled shRNA (*shScr*) or shRNAs against NOX4 (*shNOX4*) were cultured under normoxia for 24 h. At 24 h, the medium was changed and the cells were treated with 50 μM As(III) for 15 min. Thereafter proteins were harvested and subjected to Western analysis with antibodies against phospho-ERK1/2, phospho-Akt, NOX1, NOX4, and Golgi membrane (*GM*). **f** Cells were cultured as in **e**; but after 24 h, they were pre-treated for 30 min with 20 μM of the PI3K inhibitor LY294002 or the MEK inhibitor U0126, before treatment with 50 μM As(III). Proteins were harvested after 15 min for the respective blots. One hundred micrograms of total protein was subjected to Western blot analysis with antibodies against phospho-ERK1/2, phospho-Akt, Nrf2, and Golgi membrane (*GM*)

The surprising involvement of the redox-sensitive transcription factor Nrf2 in the As(III)-dependent induction of HIF-1α raised the question whether alterations in the levels of ROS contribute to the As(III)-dependent HIF-1α induction. Indeed, As(III)-treated cells displayed an enhanced ROS formation (Fig. 6a).

NADPH oxidases (NOX) have a fundamental role in ROS generation, and in particular, NOX4 appears to be involved in stimulus-dependent ROS formation. Therefore, we investigated the possible involvement of NOX4 in the induction of ROS and the HIF-1α response to As(III). To do this, we used cells in which we depleted NOX4 by specific shRNA. The results demonstrate that NOX4 is involved in the induction of ROS in response to As(III) (Fig. 6a). In addition, and as shown earlier [27, 28], we found that NOX4 is induced by hypoxia. However, treatment of cells with As(III) induced NOX4 only under normoxia (Fig. 6c, d). In line, NOX4 depletion reduced HIF-1α induction in response to As(III). As expected and in agreement with earlier data [23], NOX4 depletion also reduced hypoxia-dependent HIF-1α induction while it did not affect HIF-1α levels under combined treatment (Fig. 6b, c). In order to address whether the NOX4 effects on HIF-1α are mediated at least in part by Nrf2, we examined Nrf2 activity along with HIF-1 activity upon depletion of NOX4. We found that knockdown of NOX4 reduced induction of HIF-1α activity by As(III) and hypoxia (Fig. 6d). Interestingly, depletion of NOX4 was without effect on the Nrf2 activity under hypoxia while it reduced the As(III)-mediated Nrf2 activity under normoxia to about the same levels as observed under hypoxia (Fig. 6d). Together, these data indicate that NOX4 is involved in the As(III)-dependent regulation of HIF-1α by regulating Nrf2 activity.

It is well known that the ERK1/2 MAPK pathway and the PI3K/Akt pathway are major downstream mediators in the NOX signal chain in response to non-hypoxic stimuli [29]. Since As(III) appeared to act via NOX4, we further investigated whether the ERK1/2 and the PI3K/Akt pathways which are able to also activate Nrf2 [30] participate in the transduction of the As(III) effects. We found that As(III) treatment lead to phosphorylation of ERK1/2, and Akt; however, only depletion of NOX4 but not of NOX1 abolished ERK1/2 and Akt phosphorylation (Fig. 6e). No effects were noted under hypoxia (data not shown). In addition, we used specific inhibitors of the PI3K/Akt pathway and the ERK1/2 pathway and found that the PI3K inhibitor LY294002 abolished not only phosphorylation of Akt but also that of ERK1/2. By contrast, the MEK inhibitor U0126 only abolished the As(III)-dependent activation of ERK1/2 suggesting that phosphorylation of Akt is the upstream event necessary for the activation of ERK1/2 in response to As(III). In line, both inhibitors reduced the As(III)-induced increase in Nrf2 (Fig. 6f). Together, these data indicate that As(III) contributes to the regulation of HIF-1α via activation of NOX4, the PI3K/Akt and ERK1/2 pathway, and Nrf2.

### As(III) induces proliferation, angiogenesis, and colony formation via HIF-1α and Nrf2

**Fig. 7:**
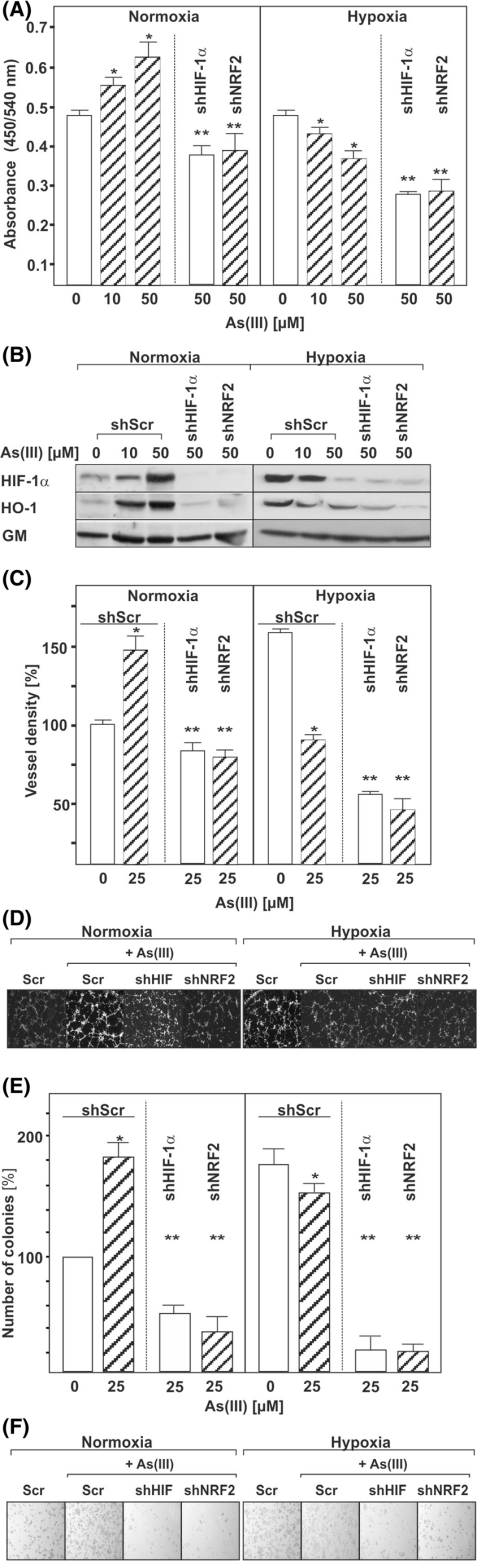
Influence of As(III) on cell proliferation. **a** HepG2 cells were cultured in 96-well plates under normoxia for 24 h. At 24 h, the medium was changed and the cells were transfected with plasmids expressing control scrambled shRNA (*shCtr*) or shRNA against HIF-1α or Nrf2. The next day, cells were cultured in the absence or presence of As(III) under normoxia (16 % O_2_) or hypoxia (5 % O_2_). After 4 h, BrdU assay was performed. Values represent mean ± SEM of at least three independent experiments. *Single asterisk*, significant difference control vs. As(III). *Double asterisks*, significant difference As(III) vs. As(III) + shNrf2 or As(III) + shHIF-1α. **b** Representative Western Blot. One hundred micrograms of total protein was subjected to Western blot analysis and probed with an antibody against HIF-1α, HO-1, or Golgi membrane (*GM*). **c** HMEC-1 cells were transfected with vectors for control scrambled shRNA (*shScr*) or shRNA against HIF-1α or Nrf2. Cells were plated onto matrigel-coated wells for 2 h and then exposed to As(III) under normoxia or hypoxia for 4 h. The number of tubules from each well was counted using ImageJ software. Data represent the number of tubules relative to the control which was set to 100 %. *Single asterisk*, significant difference shScr vs. As(III) + shScr. *Double asterisks*, significant difference As(III) + shScr vs. As(III) + shNrf2 or As(III) + shHIF-1α. **d** Photographs from a representative in vitro angiogenesis experiment. **e** Hep3B liver carcinoma cells were transfected as above. Cells were seeded onto six-well plates, exposed to As(III), and grown for 4 days. Data are presented as sum of colony volume (Σ(V)) and sum of area (Σ(A)), respectively, relative to the control which was set to 100 %. *Single asterisk*, significant difference shScr vs. As(III) + shScr. *Double asterisks*, significant difference As(III) + shScr vs. As(III) + shNrf2 or As(III) + shHIF-1α. **f** Photographs from a representative colony formation assay

We next determined whether the As(III)/Nrf2-mediated HIF-1α regulation is involved in cell proliferation. To test this, we used cells in which Nrf2 or HIF-1α were knocked-down and measured the impact of As(III) on BrdU incorporation into newly synthesized DNA. We found that under normoxia, As(III) increased cell proliferation by about 30 % in scrambled shRNA transfected control cells while Nrf2 or HIF-1α knockdown abolished the As(III) effect (Fig. 7a, b). By contrast, As(III) decreased cell proliferation under hypoxic conditions and knocking down HIF-1α or Nrf2 augmented these effects (Fig. 7a, b).

Since HIF-1α and Nrf2 are associated with the regulation of angiogenesis and tumor cell colony formation [31], we next tested whether As(III) affects capillary formation in endothelial cells and tumor colony growth of hepatoma cells in a HIF-1α or Nrf2-dependent manner using an in vitro matrigel assay and colony formation assay. Interestingly, As(III) induced both the formation of capillary-like structures and colonies under normoxic conditions. Knockdown of either Nrf2 or HIF-1α decreased formation of capillary-like structures as well as tumor cell colonies. By contrast, when capillary- and colony formation was assessed under hypoxia, it was found that As(III) reduced the hypoxia-dependent generation of capillary-like structures and colonies (Fig. 7c–f). Together, these data indicate that the regulation of HIF-1α and Nrf2 by As(III) contributes to the angiogenic responses of endothelial cells and the colony-forming properties of hepatoma cells.

## Discussion

In this study, we identified a novel mechanism by which As(III) can induce HIF-1α and contribute to proliferation and angiogenesis. Thereby, As(III) exerts effects on HIF-1α via Nrf2-mediated enhancement of transcription which is connected to ROS generation involving NOX4, stimulation of the PI3/Akt and ERK1/2 pathways as well as by increasing the HIF-1α half-life.

Although previous reports showed that As(III) can act as inducer of HIF-1α [20, 32], those studies were only focusing on partial aspects of the participating signal chains. Our study confirms several mechanistic aspects but is the first identifying two novel participating entities, namely the NADPH oxidase NOX4 and the transcription factor Nrf2.

The generation of ROS in response to As(III) was already described earlier [33] and the current study confirmed this in the hepatoma cell line HepG2. However, this phenomenon is not restricted to hepatocyte-derived cells as in this study but was also reported to occur in various other cell types [34–36]. Furthermore, earlier findings suggested that a thiol-sensitive mechanism contributes to the As(III)-induced VEGF and HIF-1α expression in human ovarian cancer cells [18].

In line with these findings and the knowledge that HIF-1α can be regulated by ROS (reviewed in [37–39]), it was tempting to investigate whether the ROS were generated due to the action of NADPH oxidases. Earlier reports have given hints that NOX proteins contribute to arsenite-induced ROS production [40–43]. However, due to the use of non-selective inhibitors like diphenyleneiodonium chloride (DPI) [44], it was so far not possible to identify the proper participating NOX member. The present study closes this gap; by using a specific knockdown approach, we were able to show that NOX4 is the predominant enzyme participating in As(III)-dependent ROS generation and HIF-1α induction. Our earlier findings [45, 46] and reports from other groups on ROS-dependent HIF-1α regulation [47–49] showed that the PI3K/Akt and the ERK1/2 MAPK pathways are of importance. Accordingly, we examined the involvement of both pathways and found that NOX4 acts upstream of the PI3K/Akt pathway and the ERK1/2 pathway, respectively. The latter findings are to some extent in line with earlier data reporting that activation of the Akt pathway contributes to the As(III)-increased HIF-1α level [20, 50, 51], though there was also a report which challenged the participation of the Akt pathway [44]. Although activation of ERK1/2 signaling was reported earlier to participate in the As(III)-mediated expression of angiogenesis-related factors and induction of HIF-1α [52, 53], our study is now first to show that the PI3K/Akt pathway and ERK1/2 pathways are interlinked and that the PI3K/Akt pathway acts upstream of ERK1/2.

In addition, we show for the first time that As(III) induced upregulation of HIF-1α mRNA under normoxia. The usage of cells with knockdown for the redox-sensitive transcription factor Nrf2 indicated that this factor plays a crucial role in the As(III)-dependent HIF-1α transcriptional regulation. Moreover, we showed that both Nrf2 and HIF-1α are participating in the As(III)-driven proliferation and angiogenic response. Indeed, similar links between HIF-1α and Nrf2 already appear to exist. For example, Nrf2 blockade seems to be of primary importance for tumors of the digestive tract. In particular, Nrf2 blockade suppressed colon tumor angiogenesis by inhibiting HIF-1α [54] and migration and invasion of esophageal squamous cell carcinoma cells in a hypoxic microenvironment [55]. In liver, it was found that Nrf2 contributes to ischemia-induced hepatocellular damage [56]. However, the action of the Nrf2 connection is not restricted to the digestive tract since knockdown of Nrf2 in glioma cells suppressed glioblastoma angiogenesis in vitro and in xenotransplanted mice by inhibiting HIF-1α [31, 57].

Vice versa, Nrf2 activation improved cell survival during hypoxia and hypoxia/reoxygenation in cardiomyoblasts [58], although in this study Nrf2 protein and activity were not induced by hypoxia.

Further, and in line with our study, the link between Nrf2 and NOX4 was also evident in cardiomyocytes [59]. However, the participation of NOX4 in the As(III) effects does not preclude that other NOX isoforms may also regulate the Nrf2-HIF-1α system. It is possible that, dependent on the cell-specific expression of the NOX proteins (for review, see [37]), other NOX isoforms may have similar effects. In that respect, an earlier study was able to show that the induction of the Nrf2 pathway in NOX1-expressing adenocarcinoma A549 cells augments HIF-1α signaling [60]. By contrast, also a feedback loop in which HIF-1 induction attenuates Nrf2-dependent gene expression appears to exist [61] indicating the complexity of the regulatory mechanism.

The connection between ROS, hypoxia, NOX4, Nrf2, and HIF-1α seems to play an important role in fibrosis where hypoxia is a major sign due to loss of endothelial cells, rarefication of capillaries, and malperfusion. In line with that, it was found that NOX4-deficient mice exhibited attenuated angiogenesis [62] and targeting NOX4-Nrf2 could reverse persistent fibrosis in lungs of aging mice [63].

**Fig. 8:**
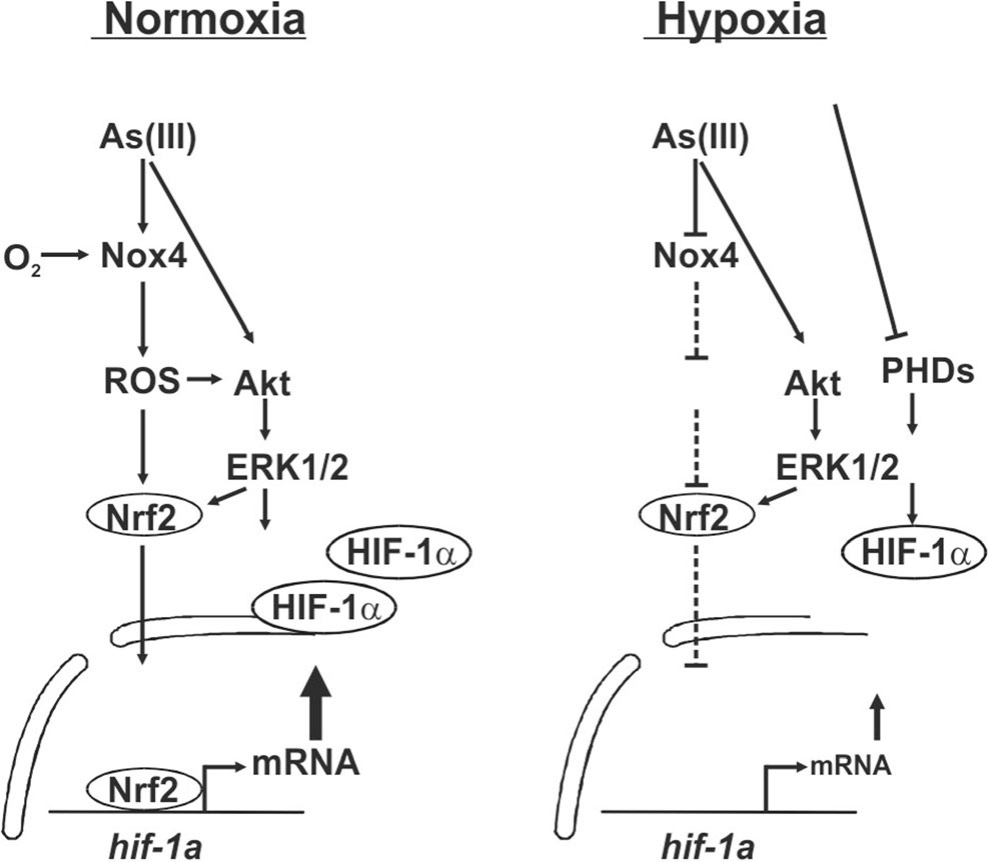
Scheme of the differential transcriptional regulation of *hif-1α* by arsenite under normoxia and hypoxia: role of Nrf2. When O_2_ is not a limiting factor, i.e., under normoxia, As(III) induces HIF-1α mRNA transcription, HIF-1α protein synthesis, HIF-1α target gene expression as well as cell proliferation and angiogenesis by involving ROS formation via NOX4 and the redox-sensitive transcription factor Nrf2. Thereby, NOX4 regulated activation of PI3K/Akt and ERK1/2 appear also to contribute to the stimulating effect. Under O_2_ limiting conditions, i.e., hypoxia, less ROS can be formed via NOX4 with the consequence that HIF-1α can no longer be efficiently transcribed due to diminished Nrf2 activity. Although the cells try to compensate against this inhibitory effect on HIF-1α transcription by prolonging its protein half-life, cell proliferation and angiogenesis are strongly impaired

In addition to the transcriptional induction by As(III) under normoxia, we could also detect that As(III) downregulated the HIF-1α mRNA levels under hypoxia which subsequently explains the decreased HIF-1α protein levels in hypoxic As(III)-treated cells. This decrease is due to the lower activity of Nrf2 under hypoxia upon As(III) treatment and although the cells tried to counteract this decreased transcription by an increase in HIF-1α protein stability, a full compensation could not be reached. The stabilization of HIF-1α in response to As(III) is primarily achieved due to the action of ROS which inhibit the action of proline hydroxylases [23, 24] and similar findings were reported by another study showing that As(III) inhibits HIF-1α degradation via ROS at the level of proline hydroxylation [44]. In view of these findings, the current data support a model where, under normoxia, As(III) induces HIF-1α mRNA transcription, HIF-1α protein synthesis, HIF-1α target gene expression as well as cell proliferation and angiogenesis via NOX4 and the redox-sensitive transcription factor Nrf2 in conjunction with activation of PI3K/Akt and ERK1/2. Under hypoxia, As(III) does not induce NOX4 so that less ROS are formed with the consequence that HIF-1α can no longer be efficiently transcribed due to diminished Nrf2 activity. To overcome this deficit, cells prolong the half-life of HIF-1α; however, the transcriptional reduction appears to be only partially compensated with the net result that the overall HIF-1α protein levels are reduced with the consequence that cell proliferation and angiogenesis are strongly impaired. Overall, the current study is the first where As(III)-dependent effects were examined under normoxia and hypoxia and where different modes of HIF-1α transcription via Nrf2 have been identified (Fig. 8).

Although our findings with HepG2, Hep3B, and HMEC1 cells support the tumor growth-promoting role of As(III), additional studies will be required to determine whether other cancer cell types respond similarly to As(III) treatment. These findings can then also be seen in the context of therapeutical use. Since HIF-1α and tumor hypoxia are considered to be associated with tumor progression [64], it would be beneficial to downregulate HIF-1α under hypoxic conditions as seen in the current study. Indeed, these findings may help to explain why arsenic is of therapeutical use; it gained a renaissance based on recent reports from China describing that a traditional Chinese medicine, “Ai-ling 1,” in which arsenic is the active ingredient, can be used to successfully treat patients with acute promyelocytic leukemia (APL) [65–67]. A number of clinical trials using arsenic to treat patients with APL confirmed the beneficiary effects [68, 69] with the consequence that arsenic has been approved as a standard therapy for the treatment of patients with relapsed or refractory APL in the USA and Europe [70] *EU/1/02/204/001;* http://ec.europa.eu/enterprise/pharmaceuticals/orphanmp/index.htm.

Overall, these findings and the results of the present study indicate that As(III) has an ambivalent mode of action with having profound effects on cell growth and angiogenesis which are associated with diseases like cancer as well as having use as therapeutic agent. The current study adds new knowledge to better understand the pro-carcinogenic mechanisms of As(III), and at the same time it aims to further foster the discussion and research to unravel the mechanisms behind the anti-carcinogenic actions of As(III).
